# The Effectiveness of Mental Health Rehabilitation Services: A Systematic Review and Narrative Synthesis

**DOI:** 10.3389/fpsyt.2020.607933

**Published:** 2021-01-13

**Authors:** Christian Dalton-Locke, Louise Marston, Peter McPherson, Helen Killaspy

**Affiliations:** ^1^Division of Psychiatry, University College London, London, United Kingdom; ^2^Department of Primary Care and Population Health, University College London, London, United Kingdom; ^3^Camden and Islington National Health Service Foundation Trust, London, United Kingdom

**Keywords:** mental health, rehabilitation, services, effectiveness, systematic review

## Abstract

**Introduction:** Mental health rehabilitation services provide essential support to people with complex and longer term mental health problems. They include inpatient services and community teams providing clinical input to people living in supported accommodation services. This systematic review included international studies evaluating the effectiveness of inpatient and community rehabilitation services.

**Methods:** We searched six online databases for quantitative studies evaluating mental health rehabilitation services that reported on one or both of two outcomes: move-on to a more independent setting (i.e. discharge from an inpatient unit to the community or from a higher to lower level of supported accommodation); inpatient service use. The search was further expanded by screening references and citations of included studies. Heterogeneity between studies was too great to allow meta-analysis and therefore a narrative synthesis was carried out.

**Results:** We included a total of 65 studies, grouped as: contemporary mental health rehabilitation services (*n* = 34); services for homeless people with severe mental health problems (*n* = 13); deinstitutionalization programmes (*n* = 18). The strongest evidence was for services for homeless people. Access to inpatient rehabilitation services was associated with a reduction in acute inpatient service use post discharge. Fewer than one half of people moved on from higher to lower levels of supported accommodation within expected timeframes.

**Conclusions:** Inpatient and community rehabilitation services may reduce the need for inpatient service use over the long term but more high quality research of contemporary rehabilitation services with comparison groups is required.

**Review registration:** This review was prospectively registered on PROSPERO (ID: CRD42019133579).

## Introduction

Most people who develop psychosis recover, but around 20% have more complex problems that require input from mental health rehabilitation services (1). The majority of this group have a diagnosis of schizophrenia, have been in contact with mental health services for many years and have had multiple acute psychiatric admissions (2).

The National Institute for Health and Care Excellence (NICE) recently published guidance on the rehabilitation of adults with complex psychosis (3). People with complex psychosis have symptoms that are persistent over a longer period and are resistant to usual treatments, they experience difficulties managing everyday activities and are likely to have additional mental and physical health comorbidities that complicate their recovery. The guideline recommends that specialist rehabilitation services should be provided for this group that includes inpatient rehabilitation and community rehabilitation teams providing specialist clinical input to people living in supported accommodation. These components should be organized into a rehabilitation care pathway and work together to support people to achieve their optimal level of independence.

Most research in this field has evaluated individual service components rather than the whole pathway. This includes two national programmes conducted in England. The REAL project (Rehabilitation Effectiveness for Activities for Life) focused on inpatient rehabilitation services and included a large cohort study. At 12-month follow-up, the majority of patients had been successfully discharged (55%) or were ready for discharge but awaiting a vacancy in supported accommodation (14%). The median length of admission in the rehabilitation unit was 16 months (4). The QuEST (Quality and Effectiveness of Supported Tenancies for people with mental health problems) project included a large cohort study that investigated outcomes for a nationally representative sample of people using mental health supported accommodation services. Over 30 months, 38% progressed from higher to lower supported settings (5). One rare example of a study investigating more than one component of the rehabilitation pathway was conducted by Killaspy and Zis [6]. This was a retrospective case note review of 141 patients of either local inpatient rehabilitation services or supported accommodation services in one NHS Trust. Over a 5-year period, they found 17 (12%) died and, of the remaining 124, 50 (40%) progressed along the rehabilitation pathway successfully (i.e., discharged from the inpatient rehabilitation unit to supported accommodation or moved from higher to lower supported accommodation services), 33 (26%) remained in supported accommodation providing the same level of support, while 41 (33%) moved “backwards” in the pathway (i.e., were admitted to hospital or moved to more supported accommodation) and only 10% of the cohort achieved fully independent living (6). Another study using the national Danish case register investigated people who moved to a registered supported accommodation service (7). They did not report move-on to more independent settings, but they did report inpatient service use and found that it reduced after the move to supported accommodation. They also found a diagnosis of schizophrenia was the strongest predictor of moving to a supported accommodation service. In summary, these findings suggest that most people using rehabilitation services stabilize and progress toward more independent settings, but a substantial proportion require longer term support.

There have been no systematic reviews of studies evaluating all components of the mental health rehabilitation pathway, but a recent review investigated the effectiveness of mental health supported accommodation (8). It categorized the included studies into three types: those evaluating deinstitutionalization programmes (studies examining the outcomes for people discharged from long term hospital admission to specialist community services); studies evaluating services for homeless people with severe mental health problems; and studies of services for people with complex longer term mental health problems who were not homeless. The strongest evidence was for services designed for the homeless population, most of which evaluated the “Housing First” approach. Unlike other supported accommodation systems, where people progress from higher to lower supported settings after demonstrating adequate ability in independent living skills (the “train and place” approach), Housing First provides people with a permanent tenancy straight away, alongside intensive, flexible support from a visiting community team.

This systematic review aimed to evaluate the international quantitative evidence for the effectiveness of mental health rehabilitation services, including hospital-based inpatient rehabilitation units, community-based rehabilitation units, community rehabilitation teams and supported accommodation services. We did not aim to review the evidence for specific psychosocial interventions that may be delivered by these services since many of these already have an established evidence base and are not necessarily delivered by rehabilitation services exclusively. Rather, we were interested in evidence for the effectiveness of the complex intervention known as mental health rehabilitation.

## Methods

### Inclusion Criteria

This review included quantitative studies in the English language that reported on at least one of two important outcomes: (1) inpatient service use, and (2) move-on from the rehabilitation service to another setting. We selected these two outcomes as they are objective measures of the effectiveness of the rehabilitation pathway and have been used in previous studies of these services (4–6). The inclusion and exclusion criteria were designed using the PICOS framework (9).

#### Population

We included studies of adults with a diagnosis of a severe mental health problem, including schizophrenia, schizoaffective disorder, and bipolar disorder. We focused on these diagnostic groups as the vast majority of users of mental health rehabilitation services have one of these as a primary diagnosis (3). We excluded studies that focused on participants with first episode psychosis (as they were unlikely to be at the stage in their illness where they had developed long term problems requiring rehabilitation), organic psychosis, substance induced psychosis, dementia, personality disorder, depression or anxiety. We included studies where more than 49% of participants had one of the included diagnoses, and where the mean age of the sample was between 18 and 65.

#### Intervention

The term “rehabilitation” has been used to describe a wide range of services and interventions in mental health. For the purpose of this review we considered a mental health rehabilitation service to be one that provided longer term care (at least 6 months) to individuals with longer term and complex mental health problems, was staffed by a multidisciplinary team (three or more disciplines), and used a biopsychosocial and person-centered approach that aimed to enable the person to gain skills for independent living and community integration. Our definition included hospital and community based rehabilitation units, community rehabilitation teams and supported accommodation services, as these include the components of a local rehabilitation pathway as recommended by NICE (3). We excluded studies that solely evaluated community services delivering assertive community treatment or intensive case management on the basis that these approaches tend to focus on people living in independent rather than staffed/supported accommodation and these models of care have been extensively evaluated (10).

#### Comparison

We did not use any inclusion or exclusion criteria relating to the type of comparison carried out in the study.

#### Outcomes

We included studies which reported on inpatient service use and/or move-on to other settings. Move-on included discharge from the rehabilitation unit to the community or from a supported accommodation service to different accommodation. Where available, we extracted the setting (type of accommodation) the individual was discharged to or moved on to.

#### Study Design

All quantitative studies were eligible, including prospective and retrospective observational studies, quasi-experimental studies and randomized controlled trials (RCTs) published since 1 January 2000. This date was selected to ensure a focus on studies investigating contemporary mental health rehabilitation services. Qualitative studies and case studies were excluded.

### Search Strategy

We searched six online databases: CINAHL Plus, EMBASE, MEDLINE, PsycINFO, The Cochrane Library and Web of Science, using subject terms and free text searches relevant to the review population (e.g., “severe mental illness”, “psychosis”, “schizophrenia”), intervention (e.g., “rehabilitation” and “supported accommodation”) and outcomes (e.g., “admission”, “readmission”, “move-on”, “discharge”). The search strategy was developed by CDL and finalized after review by HK and LM. The searches were carried out on 14 June 2019 and the results exported to EndNote (version 19.2) for de-deduplication. The searches were updated on 9 July 2020.

The titles and abstracts of all studies were screened in parallel. The full texts of studies included after this stage were then screened for final inclusion. The screening was carried out by CDL and 10% of articles at both the title/abstract and full text stages were independently screened by PM. Discrepancies were discussed, and any that could not be resolved were adjudicated by HK. Forward and backward citation searches were carried out on all studies included after the full text screening. The full search strategy is available as a Supplementary Material.

### Data Extraction

A data extraction form was used to collate data from all the included studies. We extracted meta data and other relevant details, including the year the study was published, the country where it was carried out, study design and sample selection method. We also recorded the study setting and categorized it as: (1) inpatient rehabilitation unit; (2) community rehabilitation unit; (3) community rehabilitation team; (4) supported accommodation service. We extracted data relevant to the review outcomes, including the size of the sample, the follow-up period, the number with completed follow-up, psychiatric hospitalizations and move-on to other settings. Where reported or where it could be derived, the ratio and percent of participants with a specific outcome (e.g., the proportion of participants who moved to a more independent setting or who had a hospitalization during the follow-up period) was recorded.

### Quality Assessment

We used Kmet's standardized quality assessment criteria to assess all the included studies (11). We selected this tool because it can be used with quantitative studies using various study designs. It includes 14 criteria for RCTs and 11 criteria for non-RCTs, each being scored as meeting the criterion fully (2), partially (1) or not at all (0). The scores for each item are summed, divided by the total possible score and multiplied by 100 to produce a linear score out of 100. Two researchers, the lead author (CDL) and a PhD student (SL), independently assessed a randomly selected 10% of included studies, compared and discussed their ratings and differences before independently assessing a second set of studies, again a randomly selected 10%. The agreement rate on the second set was 91% (89/98 ratings). The remaining 80% of included studies were then assessed by CDL.

### Data Synthesis

Being discharged from an inpatient rehabilitation service to the community or, for people in community supported accommodation services, moving from higher to lower levels of supported accommodation, are markers of successful rehabilitation. However, remaining at the same level of supported accommodation is an indicator of stability and can also be regarded as a positive outcome, albeit a less positive one than a move to a lower level of supported accommodation. We therefore planned to conduct meta-analyses on the following three outcomes:

1. Positive move-on (number of people who moved to a more independent setting during the follow-up period as a proportion of the total number followed-up).
2. Maintained community placement (number of people during the follow-up period who either stayed at the same community placement, moved to a setting with a similar level of support, or moved to a more independent setting, as a proportion of the total number followed-up).
3. Hospitalization (number of people who were hospitalized during follow-up as a proportion of the total number followed-up).

Most of the included studies were observational in design and reported the review outcomes as frequencies and/or proportions. To pool these proportions we used the “metaprop_one” command in Stata 14 (12), with a random-effect model. However, heterogeneity, calculated using the *I*^2^-test (13), was high (i.e., > 50%) (14), and so it was not possible to pool results, as the diversity of the results from the included studies would result in a meta-analysis estimate between those of the actual study estimates and would not give an accurate summary of results. We examined the studies by length of follow-up, to see whether this was a source of heterogeneity, but high levels of heterogeneity persisted. There are several other possible explanations for this heterogeneity, including variation between the studies in quality score, study design, and the different healthcare systems operating in the countries where included studies were conducted. We therefore proceeded by carrying out a narrative synthesis following the guidelines by Popay et al. (15).

First, we carried out a preliminary synthesis of the included studies focussing on the type of service studied and the remit of the service. Next, we explored consistencies in the results between studies, with consideration of the study design, country, sample size, follow-up period and quality assessment score (greater emphasis was placed on larger, higher quality studies). Finally, we reviewed the robustness of the synthesis by checking the main findings and the strength of these findings.

### Review Registration

This review was prospectively registered on PROSPERO (ID: CRD42019133579).

## Results

**Figure 1:**
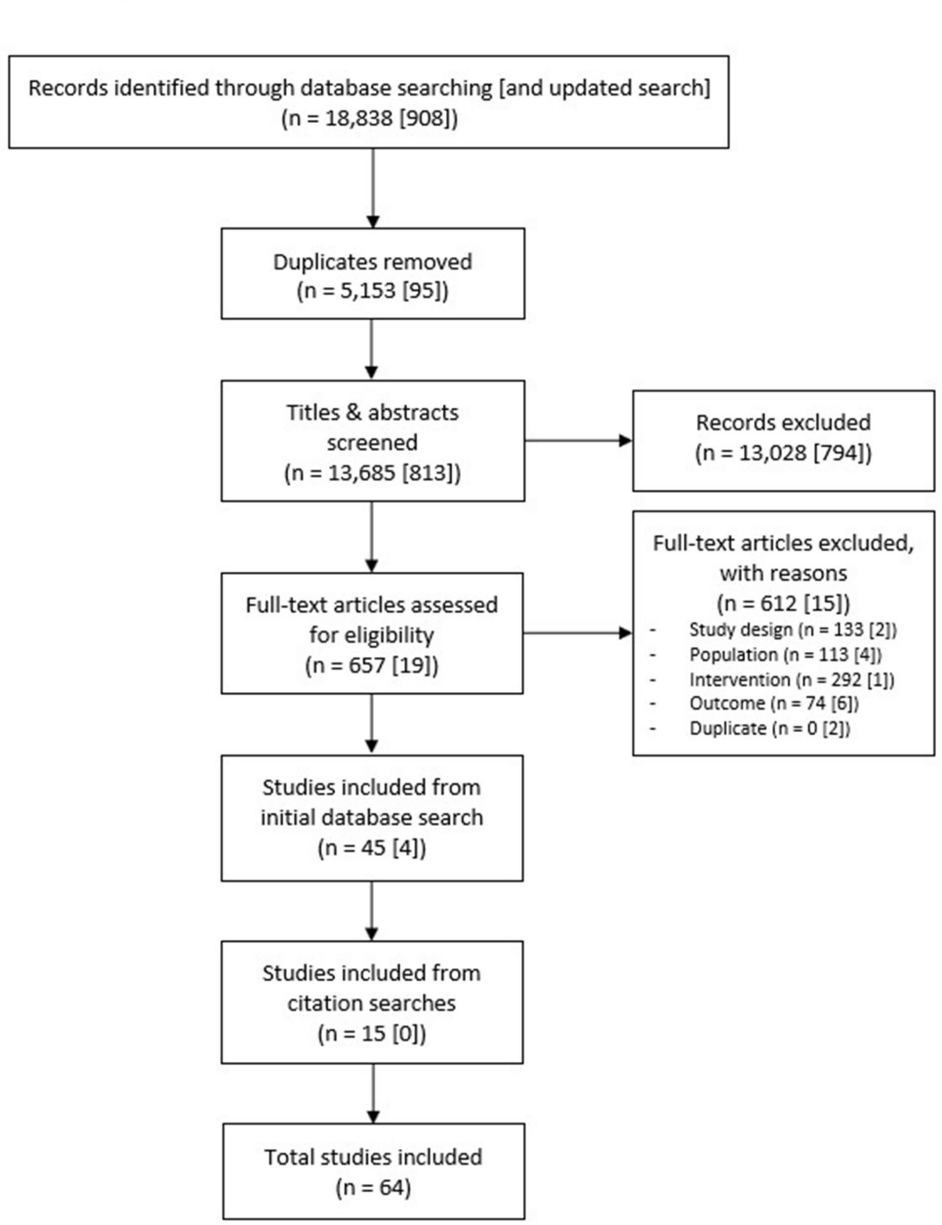
PRISMA flow diagram.

The initial database searches returned a total of 13,685 studies after de-duplication. Following screening of titles and abstracts, 13,028 studies were excluded. The full texts of the remaining 657 studies were screened, of which a further 612 were excluded, almost half (292) because they did not adequately describe the service or intervention, or because it was not a rehabilitation service. The number of studies included from the initial database searches was therefore 45. An additional four studies were included following the updated database search in July 2020, and 15 further studies were included following screening of reference lists and citations of the 49 included studies, producing a final total of 64 included studies. Figure 1 shows the number of studies at each stage.

The studies were conducted in 14 different countries: 24 in Europe (eleven UK, four Italy, three Netherlands, three Denmark, and one each in Ireland, Spain and Sweden), 19 in the United States (US), seven in Australia, five in Japan, four in Canada, three in Israel and one each in Singapore and Turkey. The vast majority were observational in design (24 prospective and 25 retrospective) and the remainder were randomized controlled trials (15). The mean quality score was 78.4 (SD 16.1). The lowest score was 40.9 and the highest was 100 (scored by 11 studies).

In regards to settings, 20 studies evaluated inpatient rehabilitation services (11 hospital based and nine community based), eight studies investigated community rehabilitation teams, 35 studies investigated supported accommodation services (one of which also investigated inpatient rehabilitation units and community rehabilitation units), and one study investigated outcomes for people who had used a rehabilitation service without specifying the setting.

The included studies could be broadly categorized as evaluating services with one of three remits. The first category, and largest in terms of the number of studies included with more than half the total (16), comprised studies investigating contemporary rehabilitation services. These were services designed for people with complex and longer term mental health problems with the specific aim of supporting them to live in more independent settings. The second category were studies investigating services for people who were homeless and had a severe mental health problem. There were 13 of these studies, all of which were conducted in the US or Canada. The last category accounted for 18 of the included studies and focused on deinstitutionalization programmes or services designed to provide a less institutional setting for patients discharged from long stay hospitals. These studies were mainly published prior to 2010. Supplementary Table 1 shows details of all the included studies, including the category as just described, country, setting, study design, review outcomes and quality score.

### Studies of Contemporary Mental Health Rehabilitation Services

This group of studies was the most varied in regards to setting and findings. Of the 33 studies in this category, two investigated community rehabilitation units, six investigated inpatient rehabilitation units, nine investigated community rehabilitation teams, 15 investigated supported accommodation services and one study did not specify the type of rehabilitation service investigated. Twenty-seven of the 33 studies were conducted as observational studies and were based in countries with different healthcare systems (eight US, seven UK, three Denmark, three Italy, two Australia, two Israel, one each in Canada, Ireland, Japan, Netherlands, Singapore, Spain, Sweden and Turkey). The mean Kmet quality score was 82.5 with eight studies scoring 100. Supplementary Table 1 provides more details regarding these studies, including a brief description of the aim of the study and relevant outcomes for our review.

The most consistent positive outcome was an improvement in inpatient service use for patients after they had an inpatient rehabilitation admission. Bunyan et al. (16), a study with a high quality score (100), compared hospital days 1-year before admission and 1-year after discharge for 501 patients from five community based rehabilitation units in Australia. The mean hospital days reduced from 101.54 (SD 113.01) before the rehabilitation admission to 70.39 (SD 118.33) afterwards. Similar findings were reported by Bunyan et al. (16) investigating a hospital based inpatient rehabilitation unit in London (16), and studies evaluating a Canadian inpatient rehabilitation unit (17) and a US rehabilitation programme (18).

A few studies reported length of inpatient rehabilitation admission before successful discharge, but the findings were inconsistent. Killaspy et al. (4) conducted a large, high quality (quality score: 95.5) cohort study involving 50 rehabilitation units across England and 339 patients, and found that most (55%) had been discharged without subsequent readmission or community placement breakdown at 12-month follow-up. Three smaller studies (*n* = 43, two inpatient units in England; *n* = 50, one inpatient unit in Ireland; *n* = 20, one inpatient unit in England) with lower quality scores (81.8, 83.3, and 59.1), reported variable rates of successful discharge: 88% at 12-month follow-up (17), 38% at 5-year follow-up (18) and 60% at 6.5-year follow-up (19).

Studies evaluating supported accommodation have reported good outcomes in terms of reduced inpatient service use. Nordentoft et al. (7) (quality score: 95.5) used the Danish national health register to investigate inpatient days for people before and after a move to supported accommodation and found a large reduction (mean 167 days in the year prior to move vs. 27 days in the year after). However, the authors were critical of the quality of care provided in supported accommodation and described these services as the “new asylums in the community” (p. 1251), with poorly defined treatment, variable staffing levels and a similar cost per day to long-stay hospitals. However, this study did not formally assess the quality of care of these services. Concerns about the content of care in supported accommodation services were also made by Anderson et al. (19) (quality score: 77.3), who found only half the residents in their sample received interventions other than medication. This was however one of the older studies published (2001) and may not be representative of current services or of services beyond the studied sample. Four other studies also reported reduced inpatient service use after a move to a supported accommodation service (quality scores: 63.6, 68.2, 72.7, 95.5) (20–23).

Cohort studies of users of mental health supported accommodation have shown that move-on to lower levels of support is somewhat limited, with the majority of residents requiring extended periods of support. Killaspy et al. (5) (quality score: 100) carried out a national cohort study in England involving 87 supported accommodation services and 619 clients. Over a 2–5 year follow-up period, fewer than half moved-on to more independent settings, despite most services having a remit to support people to move-on within 2 years. This rate differed according to the three main types of supported accommodation. Residential care provided the most intensive level of support with 24-hours staffing and daily necessities such as meals and medication catered for and here, 10% (15/146) of clients moved on. Supported housing services, with staff on-site up to 24 hours a day, had a stronger emphasis on enabling clients to gain skills for independent living and around one third (96/244) of clients moved on. Floating outreach services provided less intensive, visiting support to clients living in their own independent tenancies. Staff visited weekly on average to provide practical assistance with managing the tenancy and mental health support. Around two-thirds (132/196) of floating outreach clients moved-on over the 30 months. After taking account of differences in clinical characteristics of clients of the three types of supported accommodation, the adjusted odds ratio for move-on from floating outreach compared to residential care was 7.96 (95% CI 2.92–21.69) and 2.74 (95% CI 1.01–7.41) when compared to supported housing.

Limited move-on from supported accommodation was also found in studies based in Italy (quality scores: 100, 81.8, 86.8) (24–26), the state of Philadelphia in the US (quality scores: 85.0, 63.6) (27, 28) and in a single low quality study in Spain (quality score: 54.5) (29). However, despite limited “forward” moves toward greater independence, de Mooij et al. (30) (quality score: 100) found 78% of their sample of 262 people with severe mental illness changed address at least once over a 6-year period and 26% had changed address four or more times.

Four studies investigated predictors of successful move-on from inpatient rehabilitation units and/or supported accommodation. A large, high quality Israeli study (*n* = 2,842, quality score: 100) found higher self-reported quality of life amongst patients of inpatient rehabilitation services was associated with lower rates of re-hospitalization (31). Killaspy et al. found that the degree to which inpatient rehabilitation services (quality score: 100) (4) and supported accommodation services (quality score: 100) (5) adopted a recovery orientation was associated with successful discharge/move-on. They also found the promotion of people's human rights to be associated with successful move-on from supported accommodation services (5). Shorter hospitalizations prior to the period of inpatient rehabilitation have also been found to predict successful discharge (32) (quality score: 77.3).

Results of studies evaluating community rehabilitation teams (33–39) were mixed. Most investigated the effectiveness of a particular rehabilitation programme taking place in the community: Illness Management and Recovery (IMR) (35, 37, 39, 40). The IMR programme primarily comprises psychoeducation and promotion of personal recovery delivered via weekly group sessions over the course of 9 months. None of these studies found the intervention to be associated with a reduction in inpatient service use. A high quality (quality score: 100) RCT involving 198 participants comparing IMR with TAU also found no difference at 12-month follow-up in terms of functioning, symptoms or emergency room visits (37).

Four other studies we identified also investigated community rehabilitation teams, two of which were published recently (34, 36) but differed considerably in quality scores (59.1 and 100). The high quality study reviewed health records to investigate 4-year outcomes for 193 patients of an inner-city team in the UK that supported people living in 24-hour supported accommodation (34). The authors found that fewer than one-in-four (*n* = 45, 23.3%) clients moved on to more independent accommodation. The lower quality study investigated the outcomes of a case management model based on rehabilitation principles in Turkey. They found that for 30 patients, their psychiatric hospital admission rate reduced from a mean of 1.33 (SD 1.06) over a 2-year period before case management, to 0.23 (SD 0.56) over the same length of time during case management.

A very small (*n* = 8) and low quality study (quality score: 57.7) conducted in Israel examined the effectiveness of cognitive behavioral therapy (CBT) for people with a diagnosis of schizophrenia participating in a day treatment programme based on psychiatric rehabilitation principles, by randomizing them to CBT (day treatment plus CBT) or TAU (day treatment only). They found no difference in the number of hospital admissions between groups (33). A much larger study (*n* = 370) (quality score: 80.8) also conducted in Israel investigated the effectiveness of clinical case management for “revolving door patients,” which included providing training of skills necessary for daily living tasks, but failed to show it to be effective in reducing hospital admissions when compared to TAU (41).

Only one study included more than two components of the rehabilitation pathway, and was briefly described in the Background section. Killaspy and Zis (6) (quality score: 95.5) used retrospective case note review to investigate the outcomes of 141 patients of three inpatient rehabilitation units, two community rehabilitation units and four supported accommodation services, all located in two inner city London boroughs. Over 5 years, 40% of those with complete follow-up (50/124) had progressed along the rehabilitation pathway, 27% (33/124) had maintained their placement and 38% (41/124) had a “backwards” move.

### Studies of Services for Homeless People With Severe Mental Health Problems

This group of studies recruited participants that were either homeless or at risk of homelessness. The mean Kmet quality score was 83.8 with three studies scoring 100. The majority (8/13) of these studies were RCTs and all but one evaluated models of supported accommodation. The exception was a study investigating a long-term compulsory inpatient unit based in the Netherlands specifically for people who were homeless and had a treatment resistant severe mental health problem and a substance misuse problem (42). All the other studies in this category were conducted in the US or Canada and most (10/13) investigated either the “Housing First” (43–49) or “Full Service Partnership” (50–52) programme. Supplementary Table 1 provides more details regarding these studies.

The Full Service Partnership model is very similar to the Housing First approach, and Gilmer et al. describes it as a Housing First program that does “whatever it takes to improve residential stability and mental health outcomes” (p.646) (50). Gilmer et al. (quality score: 100) (52) found that Full Service Partnership programmes with higher fidelity to the Housing First model were more effective in reducing the number of days spent homeless. Low fidelity programmes resulted in a mean reduction of 34 days per year spent homeless (95% CI−55 to −13) whereas high fidelity programmes had a mean reduction of 87 days (95% CI−109 to −64).

All the Housing First (43–49) and Full Service Partnership studies (50–52) reported the approach to be effective at reducing homelessness/improving housing stability. The strongest evidence was reported by Aubry et al. (quality score: 100) (44). They carried out a multi-center RCT in Canada, allocating 950 participants to Housing First or TAU (access to all the locally available housing services, except for Housing First) and tracked their housing status and health outcomes over 2 years. At the final 2-year follow-up, 74% (95% CI = 69 to 78%) of Housing First clients were in stable housing compared to only 41% (95% CI = 35 to 46%) of those receiving TAU. Housing First clients were also housed quicker and rated their accommodation as better quality.

The two studies in this category that did not investigate Housing First or Full Service Partnership programmes also found that the model being evaluated had a positive impact on housing stability. Lipton et al. (53) (quality score: 85.0) studied the effectiveness of supportive housing in New York City. They defined the term “supportive housing” to describe all housing services with integrated support for people with a severe mental illness. At 2-year and 5-year follow-up, 64% and 50% of their 2,937 participants, respectively, were in stable housing. The other study compared a non-integrated model of care (housing and mental health support provided by two separate agencies) with an integrated approach (where the two components were provided by the same agency) and found participants randomized to the integrated approach at 18-month follow-up had spent more days in stable housing (quality score: 80.8) (54).

The one study in this category which did not evaluate a model of supported accommodation investigated a long-term compulsory inpatient ward based in the Netherlands, “Sustainable residence (SuRe),” reported the numbers of different types of discharge from the service over a 4-year period (quality score: 86.4) (42). Most of the discharges were to a less restrictive setting, including voluntary psychiatric wards and supported housing (69/165, 42%), but a minority were transferred to a more supported setting (16/165, 16%).

### Studies of Deinstitutionalization Programmes

The overall findings of the 18 included deinstitutionalization studies was that the process of closing the large institutions and discharging long stay patients to specialist community services was successful. All except one of the studies were observational; one study randomly allocated patients to continued hospitalization or to a group home (55). They were conducted in a number of different countries (five Australia, four Japan, four UK, two US, and one each in Israel, Italy and Netherlands), and had lengthy follow-ups but were generally of low quality (mean quality score: 67.1, none of the studies scored 100). Only three studies followed patients for less than 2 years post-discharge (56–58). Supplementary Table 1 provides more details regarding these studies.

Most patients were clinically stable in the community (58–64) with improvement in positive symptoms of psychosis (55, 61, 65), social functioning (55, 58, 65), and challenging behaviors (60) at final follow-up. One study reported greater improvements in social functioning and clinical symptoms in patients who were more severely unwell at recruitment (66). Importantly, patients were also more satisfied with their living arrangement in the community when compared to hospital (61, 67). Following their initial discharge to the community, a substantial proportion of patients subsequently moved to more independent settings with less than 24-hour staff supervision (60, 68–70). However, conversely, Chopra et al.'s small study of 18 people reported patients were less satisfied with their accommodation following the subsequent move, and were often still living in “restrictive” settings and unhappy about making recurrent moves (67). Two studies found older patients were less likely to do as well (57, 58). This may partly be explained or conflated with the finding that a longer stay in hospital is associated with unfavorable outcomes (71) and the fact that older patients of the institutions were more likely to have more severe, longer term mental health problems than younger patients.

Trieman et al. (64) tracked the “difficult to place” patients who were the last to be discharged from a North London asylum. At 5-year follow-up they had similarly positive outcomes to those who had been discharged earlier, including clinical stability and a reduction in challenging behavior. Many had moved on from their initial community placement to a more independent setting. Similar findings were reported by two smaller studies in the US (56, 72).

## Discussion

The 64 included studies were too heterogeneous for meta-analysis, therefore a narrative synthesis was carried out. Heterogeneity was mainly due to the broad concept of mental health rehabilitation in research but there were also differences between studies regarding the country where they were based and the health care systems which operate in these countries. To facilitate the narrative synthesis, we categorized studies based on the broad remit of the service or intervention which the study evaluated.

### The Contemporary Rehabilitation Studies

The most consistent positive finding was reduced inpatient service use after an inpatient rehabilitation admission (16, 17) or move to a supported accommodation service (7, 20–23) compared to the period before the admission/stay. However, these studies were mostly observational and only one (evaluating supported accommodation) included a comparison group (7). The findings should therefore be interpreted with caution. Given that randomized controlled trials are likely to be unfeasible (73), further studies with valid comparison groups are needed to control for possible confounders at the patient and service level.

Several studies found that people were unable to move on from supported housing within the expected timeframes (5, 24–28). This suggests that these timeframes require review and that services should be commissioned to be able to provide more flexible and individually tailored support, with the understanding that an individual may continue to require the current level of support in the longer term. This finding may also suggest that there is a lack of appropriate accommodation for people to move on to but further research measuring readiness for move-on as well as actual move-on are needed to confirm this as an explanation. The provision of more, appropriately resourced, floating outreach or Housing First services could help to address this by providing permanent accommodation for people leaving supported housing. Furthermore, the visiting support provided to people in their own homes through the floating outreach approach can be tailored according to fluctuations in the individual's needs and, if resourced appropriately, can provide an alternative to the stepped supported housing pathway that necessitates recurrent moves for people as they progress in their recovery. However, it is essential that when targeted at people with longer term and complex mental health needs, such models are combined with specialist clinical input from a community rehabilitation team able to offer intensive case management or assertive community treatment (44). It is also important that service planners acknowledge that some individuals have such high support needs that even an augmented floating outreach approach such as this will not provide adequate support. In addition, some individuals prefer to live in congregate settings with staff on-site rather than individual tenancies. A variety of supported accommodation models will therefore be required within a local area, based on the needs of the local population, as recommended by the recent NICE guideline on rehabilitation for adults with complex psychosis (3).

The recent NICE guideline recommends that local rehabilitation services should include community rehabilitation teams (3). We found few studies evaluating this model of care but consistent amongst them was the finding that the use of the Illness Management and Recovery programme did not reduce the need for inpatient services (35, 37, 39, 40). Only one study investigated the effectiveness of community rehabilitation teams with regard to supporting clients to achieve successful move on to more independent accommodation (34). Further high quality research is needed to investigate the effectiveness of these services. Other outcomes may also need to be considered given the limited number of moves to more independent settings.

### Services for Homeless People With Severe Mental Health Problems

Most of the studies of the homeless population were trials of the Housing First model conducted in North America and they all reported positive outcomes with regard to housing stability (43–52). A recent systematic review and meta-analysis supported this finding but found less clear evidence for other outcomes including mental health symptoms, substance misuse and employment (74). Indeed, the largest trial included in our review found no difference between groups in days hospitalized, number of emergency department visits, arrests or mental health symptoms, when compared to TAU (44). There is strong evidence Housing First does address homelessness amongst people with severe mental health problems, but further research is required on other outcomes and on other populations. If found effective for non-homeless people with complex longer term mental health problems, then it should be considered as a component in the mental health rehabilitation pathway.

### The Deinstitutionalized Population Studies

The deinstitutionalization studies, in the main, reported positive outcomes. Most individuals were successfully discharged from long stay hospitals to community settings without any clinical deterioration (58–65). There were however a substantial minority who required high levels of community support long term (64, 70, 71). This is in keeping with the findings from the recent cohort studies included in our “contemporary rehabilitation” group of studies that showed a relatively low rate of move-on to more independent settings and that people with higher levels of complex needs are likely to require long term supported accommodation. The success of the deinstitutionalization of mental health care is well-established, and critics who claimed that the closure of long term hospitals led to homelessness and imprisonment of people with mental health problems (75, 76) have been disproven by high quality cohort studies (77).

### Strengths and Limitations

The main strength of this systematic review is its comprehensiveness. Six online databases were searched, which returned 13,685 articles after deduplication in the original search and 813 in the updated search. This was supplemented by forward and backward citation searches of included studies. Screening and quality assessments were corroborated by a second researcher and the review was prospectively registered. Its main limitation was that the included studies covered a broad range of rehabilitation services from a number of different countries with different healthcare systems, and were, unfortunately, too heterogeneous for meta-analysis.

The term “rehabilitation” has been used in mental health to describe a range of different approaches, and depending on how the intervention has been described, it was not always possible to distinguish a mental health rehabilitation service from a general mental health service. We may therefore have excluded studies that could be relevant for our target population. Future research in this field would benefit from providing a clear description of the content of the complex intervention known as mental health rehabilitation that is being evaluated, alongside a detailed description of the people it targets. Although our review included a range of approaches to mental health rehabilitation, our outcomes were chosen as relevant markers of success across services. Nevertheless, additional outcomes beyond the scope of this review are likely to be important and useful for future research, such as improvement in functioning, and quality of life.

Our review aimed to review the quantitative evidence for mental health rehabilitation services and as such, we excluded qualitative research. We have now established that the current quantitative evidence in this field does not lend itself to meta-analyses, at least on our selected outcomes, and further reviews should therefore consider inclusion of relevant qualitative studies that may provide important contextual and experiential evidence. Finally, we did not include gray literature, trial registers or non-English language studies and therefore relevant studies from these sources would not have been identified.

## Conclusions

The field of mental health rehabilitation research is heterogenous and lacking in some areas. There is reasonable evidence to suggest that inpatient rehabilitation and supported accommodation can reduce inpatient service use for people with more complex and longer term mental health problems, but people do not move on from supported accommodation at the expected rate. The strength of these findings is limited to observational studies that for the main part do not use comparison groups. There is a lack of studies which consider the whole rehabilitation pathway. There is quite strong evidence for the Housing First model in reducing homelessness but its effectiveness in regard to other outcomes and when targeting people with complex mental health problems who are not homeless remains unclear.

## Data Availability Statement

The full search for each database is included in the Supplementary Materials. Please contact the authors for details of the articles rejected at each stage of screening. All other original contributions provided by this study are included in the article/Supplementary Material.

## Author Contributions

CD-L, HK, and LM conceived and designed the review. CD-L drafted the protocol and search strategy, which was reviewed and revised by HK, LM, and PM. CD-L screened the search returns and 10% were independently screened by PM at both the title/abstract stage and full text stage. Quality assessment of included studies were conducted by CD-L and 20% were independently assessed by SL (see Acknowledgments). CD-L drafted the article, which was reviewed and revised by all authors. All authors approved the final version of the manuscript.

## Conflict of Interest

The authors declare that the research was conducted in the absence of any commercial or financial relationships that could be construed as a potential conflict of interest.
