# Supplementary material for: The Effectiveness of Mental Health Rehabilitation Services: A Systematic Review and Narrative Synthesis
Source: Front Psychiatry. 2021 Jan 13;11:607933. doi: 10.3389/fpsyt.2020.607933 (PMC7838487; doi:10.3389/fpsyt.2020.607933)
Supplement: Supplementary file 1 [file Data_Sheet_1.PDF]

Supplementary materials 1. Search strategy

Final search<br>Ovid - MEDLINE		
LINE	*SEARCH*	*Results 13/06/2019*
1	Exp Mental Disorders/	1176937
2	exp Mental Health/	34013
3	((psycho* or psychiat* or mental*) adj (illness* or disorder* or problem* or disease* or disab* or health or well being or wellbeing)).tw.<br>*[tw= search in: title, abstract]*	217487
4	(severe mental illness or serious mental illness).tw.	5455
5	schizo*.tw.	120434
6	or/1-5	1295851
7	Hospitals, Psychiatric/	24808
8	Psychiatric Rehabilitation/	287
9	Community Mental Health Centers/	2887
10	Mental Health Services/	31879
11	((community or inpatient or in-patient or hospital or service) adj3 rehab*).tw.	10525
12	Residential facilities/	5288
13	Group homes/	947
14	Residential treatment/	3019
15	(((support* or shelter* or board* or group or resident*) adj (hous* or home* or accom* or living or lodg* or tenanc* or care or support* or rehab*)) or hostel or ((floating or visiting) adj (support or outreach))).tw.	25088
16	or/7-15	99197
17	exp hospitalization/	221518
18	Patient Discharge/	27099
19	mental health recovery/	71
20	patient readmission/	14887
21	(admission* or re?admission*).tw.	187886
22	bed adj day*.tw.	1838
23	length of stay.tw.	41893
24	(patient adj (discharge* or recovery)).tw.	2751
25	(independent) adj (accom* or living).tw.	2183
26	((less or lower) adj3 accom*).tw.	2579
27	(more adj3 accom*).tw.	2741
28	(Move on or moveon or move-on or moved on or moved-on).tw.	1338
29	or/17-28	371403
30	6 and 16 and 29	9350
31	Limit 30 to English language	8146
32	Limit 31 to yr="2000 -Current"	3884

Final search<br>Ovid - EMBASE		
LINE	*SEARCH*	*Results 13/06/2019*
1	exp *Mental Disease/	1276960
2	exp *Mental Health/	39863
3	((psycho* or psychiat* or mental*) adj (illness* or disorder* or problem* or disease* or disab* or health or well being or wellbeing)).tw.<br>*[tw= search in: title, abstract]*	337402
4	(severe mental illness or serious mental illness).tw.	8287
5	schizo*.tw.	170269
6	or/1-5	1503910
7	Mental hospital/	23137
8	psychosocial rehabilitation/	1285
9	Community Mental Health Center/	2887
10	Mental Health Service/	52247
11	((community or inpatient or in-patient or hospital or service) adj3 rehab*).tw.	19951
12	Residential home/	6719
13	Residential care/	11363
14	(((support* or shelter* or board* or group or resident*) adj (hous* or home* or accom* or living or lodg* or tenanc* or care or support* or rehab*)) or hostel or ((floating or visiting) adj (support or outreach))).tw.	45102
15	or/7-14	152673
16	exp hospitalization/	333443
17	mental health recovery/	165
18	hospital discharge/	111465
19	hospital readmission/	53312
20	admission* or re?admission*.tw.	458669
21	bed adj day*.tw.	3667
22	length of stay.tw.	92195
23	(patient adj (discharge* or recovery)).tw.	5520
24	(independent) adj (accom* or living).tw.	3518
25	((less or lower) adj3 accom*).tw.	3728
26	(more adj3 accom*).tw.	4061
27	(Move on or moveon or move-on or moved on or moved-on).tw.	2134
28	or/16-27	856989
29	6 and 15 and 28	9789
30	Limit 29 to English language	8719
31	Limit 30 to yr="2000 -Current"	6800

Final search<br>*Ovid - PsycINFO*		
LINE	*SEARCH*	*Results 13/06/2019*
1	Exp Mental Disorders/	816545
2	exp Mental Health/	60893
3	((psycho* or psychiat* or mental*) adj (illness* or disorder* or problem* or disease* or disab* or health or well being or wellbeing)).tw.<br>*[tw= search in: title, abstract]*	332392
4	(severe mental illness or serious mental illness).tw.	8518
5	schizo*.tw.	126327
6	or/1-5	1017298
7	Psychiatric hospitals/	7723
8	Psychosocial Rehabilitation/	3977
9	Community Mental Health Centers/	2746
10	Mental Health Services/	33106
11	((community or inpatient or in-patient or hospital or service) adj3 rehab*).tw.	4883
12	Residential Care Institutions/	10250
13	Group homes/	1085
14	(((support* or shelter* or board* or group or resident*) adj (hous* or home* or accom* or living or lodg* or tenanc* or care or support* or rehab*)) or hostel or ((floating or visiting) adj (support or outreach))).tw.	15543
15	or/7-14	72471
16	exp hospitalization/	21936
17	(admission* or re?admission*).tw.	38174
18	bed adj day*.tw.	304
19	length of stay.tw.	4974
20	(patient adj (discharge* or recovery)).tw.	455
21	(independent) adj (accom* or living).tw.	2862
22	((less or lower) adj3 accom*).tw.	620
23	(more adj3 accom*).tw.	1463
24	(Move on or moveon or move-on or moved on or moved-on).tw.	1519
25	or/16-24	61633
26	6 and 15 and 25	3922
27	Limit 26 to English language	3615
28	Limit 27 to yr="2000 -Current"	2258

Final search<br>*EBSCOhost – CINAHL Plus*		
LINE	*SEARCH*	*Results 14/06/2019*
1	(MM "Mental Disorders+")	373084
2	(MH "Mental Health")	30797
3	((psycho* or psychiat* or mental*) W0 (illness* or disorder* or problem* or disease* or disab* or health or well being or wellbeing))<br>*[search in: Abstract, Author, Keywords, Classification Codes, Subjects, Title and Translated Title]*	210998
4	(severe mental illness or serious mental illness)	5356
5	schizo*	29418
6	S1 OR S2 OR S3 OR S4 OR S5	503105
7	MH "Hospitals, Psychiatric"	6238
8	MH "Psychiatric Units"	2325
9	MH "Psychiatric Service"	809
10	MH "Rehabilitation, Psychosocial+"	4760
11	MH "Community Mental Health Services+"	10953
12	MH "Psychiatric Patients"	10806
13	MH "Inpatients"	75089
14	((community OR inpatient OR hospital) W3 rehab*)	6546
15	MH "Residential Care+"	6873
16	MH "Residential Facilities+"	29117
17	MH "Psychiatric Home Care"	217
18	(((support* OR shelter* OR board* OR group OR resident*) W0 (hous* OR home* OR accom* OR living OR lodg* OR tenanc* OR care OR support* OR rehab*)) OR hostel OR ((floating OR visiting) W0 (support OR outreach)))	20739
19	S7 OR S8 OR S9 OR S10 OR S11 OR S12 OR S13 OR S14 OR S15 OR S16 OR S17 OR S18	151383
20	(MH "Hospitalization+")	88127
21	(admission* OR readmission* OR re-admission* OR (bed W0 day*) OR "length of stay")	118825
22	MH "Discharge Planning+"	4641
23	MH "After Care"	11844
24	(patient W0 (discharge* OR recovery))	21358
25	((independent) W0 (accom* or living))	1994
26	((less or lower) W3 accom*)	216
27	(more W3 accom*)	380
28	(Move on or moveon or move-on or moved on or moved-on)	25446
29	S20 OR S21 OR S22 OR S23 OR S24 OR S25 OR S26 OR S27 OR S28	241689
30	S6 AND S19 AND S29	5952
31	S6 AND S19 AND S29 **Limiters** - Publication Year: 2000-2019; English Language	4916

Final search<br>*Web of Science*		
LINE	*SEARCH*	*Results 14/06/2019*
1	TS=((psycho* or psychiat* or mental*) NEAR/0 (illness* or disorder* or problem* or disease* or disab* or health or "well being" or wellbeing))<br>*[TS = search in: Title, Abstract, Author Keywords, Keywords Plus]*	344890
2	TS=(severe mental illness or serious mental illness)	16489
3	TS=schizo*	220551
4	#3 OR #2 OR #1	525724
5	TS=((community OR inpatient OR hospital) NEAR/3 rehab*)	13344
6	TS=(((support* OR shelter* OR board* OR group OR resident*) NEAR/0 (hous* OR home* OR accom* OR living OR lodg* OR tenanc* OR care OR support* OR rehab*)) OR hostel OR ((floating OR visiting) NEAR/0 (support OR outreach)))	43957
7	#6 OR #5	57133
8	TS=(admission* OR readmission* OR re-admission* OR (bed NEAR/0 day*) OR "length of stay")	241565
9	TS=(patient NEAR/0 (discharge* OR recovery))	8721
10	TS=((independent) NEAR/0 (accom* or living))	3818
11	TS=((less or lower) NEAR/3 accom*)	12302
12	TS=(more NEAR/3 accom*)	14952
13	TS=(Move on or moveon or move-on or moved on or moved-on)	366610
14	#13 OR #12 OR #11 OR #10 OR #9 OR #8	642186
15	#14 AND #7 AND #4	533
16	#14 AND #7 AND #4 **Limiters** - Publication Year: 2000-2019; English Language	410

Final search<br>*Cochrane Library*		
LINE	*SEARCH*	*Results 14/06/2019*
1	MeSH descriptor: [Mental Disorders] explode all trees	64650
2	MeSH descriptor: [Mentally Ill Persons] this term only	46
3	MeSH descriptor: [Mental Health] this term only	1282
4	((psycho* or psychiat* or mental*) NEAR/0 (illness* or disorder* or problem* or disease* or disab* or health or "well being" or wellbeing)):ti,ab,kw	86
5	(severe mental illness or serious mental illness):ti,ab,kw	2430
6	schizo*:ti,ab,kw	16750
7	#1 OR #2 OR #3 OR #4 OR #5 OR #6	75729
8	MeSH descriptor: [Hospitals, Psychiatric] this term only	237
9	MeSH descriptor: [Psychiatric Rehabilitation] this term only	32
10	MeSH descriptor: [Rehabilitation Centers] explode all trees	653
11	MeSH descriptor: [Community Mental Health Centers] explode all trees	109
12	MeSH descriptor: [Community Mental Health Services] this term only	686
13	MeSH descriptor: [Psychiatric Department, Hospital] this term only	95
14	((community OR inpatient OR hospital) NEAR/3 rehab*):ti,ab,kw	2915
15	MeSH descriptor: [Residential Treatment] this term only	153
16	MeSH descriptor: [Residential Facilities] explode all trees	1628
17	MeSH descriptor: [Group Homes] this term only	44
18	(((support* OR shelter* OR board* OR group OR resident*) NEAR/0 (hous* OR home* OR accom* OR living OR lodg* OR tenanc* OR care OR support* OR rehab*)) OR hostel OR ((floating OR visiting) NEAR/0 (support OR outreach))):ti,ab,kw	108253
19	#8 OR #9 OR #10 OR #11 OR #12 OR #13 OR #14 OR #15 OR #16 OR #17 OR #18	113181
20	MeSH descriptor: [Hospitalization] explode all trees	13019
21	MeSH descriptor: [Mental Health Recovery] this term only	3
22	(admission* or re?admission*):ti,ab,kw	5139
23	(bed NEAR/0 day*):ti,ab,kw	2
24	(length of stay):ti,ab,kw	22443
25	(patient NEAR/0 (discharge* or recovery)):ti,ab,kw	3
26	(independent) NEAR/0 (accom* or living):ti,ab,kw	0
27	((less or lower) NEAR/3 accom*):ti,ab,kw	246
28	(more NEAR/3 accom*):ti,ab,kw	238
29	(Move on or moveon or move-on or moved on or moved-on):ti,ab,kw	4205
30	#20 OR #21 OR #22 OR #23 OR #24 OR #25 OR #26 OR #27 OR #28 OR #29	35704
31	#7 AND #19 AND #30	786
32	#7 AND #19 AND #30 Limiters - Publication Year: 2000-2019, in Trials	524

Final search: Databases combined – 14/06/2019	
Database	***Results***
Medline	3923
Embase	6806
PsycINFO	2259
CINAHL Plus	4916
Web of Science	410
Cochrane	524
Total	18838
De-duplicated total	13685

Relevance check		
Author, year and title of known paper	*Paper included in results?*	*Notes*
Bunyan et al 2016: 'In-patient rehabilitation: clinical outcomes and cost implications'	YES	-
Killaspy and Zis, 2013: 'Predictors of outcomes for users of mental health rehabilitation services: a 5-year retrospective cohort study in inner London, UK'	YES	-
Killaspy et al 2016: REAL cohort study – 'Clinical outcomes and costs for people with complex psychosis; a naturalistic prospective cohort study of mental health rehabilitation service users in England'	YES	-
Lavelle et al 2007: 'Mental Health Rehabilitation and Recovery Services in Ireland: A multicentre study of current service provision, characteristics of service users and outcomes for those with and without access to these services'	NO	Paper not published in peer reviewed journals. Searched http://www.opengrey.eu/ (23/06/2019), and also does not appear in this database.
Kelbrick et al 2016: 'Evaluating outcomes in an adult inpatient psychiatric rehabilitation unit'	YES	-
Killaspy et al 2019: QuEST cohort study – 'Predictors of moving on from mental health supported accommodation in England: national cohort study'	YES	-
Trieman, N. and Leff, J. (2002): 'Longterm outcome of long-stay psychiatric in-patients considered unsuitable to live in the community: TAPS Project'	YES	-

Updated search: Databases combined – 09/07/2020	
Database	***Results***
Medline	113
Embase	214
PsycINFO	108
CINAHL Plus	294
Web of Science	75
Cochrane	104
Total	908
De-duplicated total	813

Inclusion/Exclusion criteria:	
Include:	
Study design	Quantitative studies published in peer reviewed journals.
Population	>49% sample people with longer term SMI (psychosis/bipolar).
Intervention	Care provision of intervention/service is sufficiently described and is consistent with the 'psychiatric rehabilitation' ethos. I.e. comprised of longer term (mean or median = >6mo), holistic (biopsychosocial), person-centred care based on rehabilitation principles (increased QoL, community integration, progress to greater independence / ADLs and greater autonomy). The service/intervention should specifically provide for people with longer term SMI, they may be structured using a 'case management' (care coordination) approach and work collaboratively with other healthcare services. The intervention/service should be recognisable as one of the following: an inpatient rehab unit, a community rehab unit, a community rehab team (including CMHT with rehab focus) or a supported accommodation service (residential care, supported housing, floating outreach). Studies which are trialling an intervention but using a rehab service as TAU/control.
Outcome	Psychiatric hospitalisation during/post intervention (>3month FU post hospital discharge), and/or discharge or move-on rate from intervention service, and/or tenure of community placement/residency.
Exclude:	
Study design	Qualitative studies, case reports, reviews, guidelines, protocols, commentaries, editorials, poster abstracts, conference abstracts, non-English language.
Population	People with first episode psychosis, mood/affective disorder, dementia or learning disability. Samples with mean or median = <18 or >64.
Intervention	Insufficient information. Acute (crisis) services (<6mo), forensic services, assertive community treatment/outreach / intensive case management, early intervention in psychosis, nursing homes, non-mh focus (e.g. drug/alcohol treatment).
Outcome	-
