# Supplementary material for: The Effectiveness of Mental Health Rehabilitation Services: A Systematic Review and Narrative Synthesis
Source: Front Psychiatry. 2021 Jan 13;11:607933. doi: 10.3389/fpsyt.2020.607933 (PMC7838487; doi:10.3389/fpsyt.2020.607933)
Supplement: Supplementary file 2 [file Table_1.pdf]

Supplementary materials 2. Characteristics and review outcomes of the included studies

First author, Year published	Country	Primary setting	Main aim	Design	Sample selection	N (by group)	Follow-up (FU) in months	Ratio (%) moved to higher supportive setting	Ratio (%) moved to lower supportive setting	Inpatient service use	Quality rating
**Contemporary rehabilitation studies**											
Anderson, 2001	US	Supported accom.	To characterise residents (their socio-demographics and service utilisations) of intermediate care facilities	Obs., retro.	Random selection amongst residents with schizophrenia or schizoaffective disorder	179	12	NR	NR	Proportion with an admission during FU 80/179 (45%)	77.3
Awara, 2017	Canada	Hospital rehab. unit	Describe characteristics of inpatient rehab. unit inpatients, investigate hospital days pre- and post-admission	Obs., retro.	All discharges from single inpatient unit	80	6	7/58 (12.1%)	48/58 (82.8%)	Proportion with an admission six months pre-rehab. = 48/53 (90.6%); six months post-rehab. = 10/53 (18.9%)	81.8
Blow, 2000	US	Hospital rehab. unit	Investigate effects on service utilisation of four treatment programmes: STAR-II (an intensive inpatient rehabilitation programme), day treatment aiming to support and train for living in the community, assertive community treatment (ACT), and TAU	Obs., prosp.	Patients enrolled on treatment programme	1425	36	NR	NR	Mean (SD) hospitalised days one-yr before baseline vs. one-yr before 3-yr FU:<br>STAR-II: 274.0 (101.7) vs. 149.1 (157.6);<br>Day treatment: 221.4 (102.7) vs. 74.5 (104.0);<br>ACT: 258.4 (109.4) vs. 104.2 (133.3);<br>TAU: 246.6 (136.9) vs. 185.5 (163.3)	68.2
Bota, 2007	US	Supported accom.	Does boarding home reduce inpatient use	Obs., retro.	Patients discharged from boarding home (BH)	20	37	NR	NR	Pre-BH = mean 3.3 days per month; During-BH = 0.33; Post-BH = 0.24	68.2

First author, Year published	Country	Primary setting	Main aim	Design	Sample selection	N (by group)	Follow-up (FU) in months	Ratio (%) moved to higher supportive setting	Ratio (%) moved to lower supportive setting	Inpatient service use	Quality rating
**Contemporary rehabilitation studies (continued)**											
Bradshaw, 2000	US	Community rehab. team	Investigate effectiveness of CBT for schizophrenia compared to day treatment programme (DTP)	RCT	Patients consecutively referred to day treatment programme following discharge from inpatient admission who met study criteria	24	36	NR	NR	CBT&DTP (n=8) = Yr1: Mean 5.0 (SD 7.87), Yr2: 0 (0), Yr3: 0 (0); DTP (n=7) = Yr1: 2.57 (3.82), Yr2: 2.71 (4.86), Yr3: 2.29 (4.27).	57.7
Bunyan, 2016	UK	Hospital rehab. unit	Investigate clinical and economic effectiveness of inpatient rehab. units	Obs., retro.	Sequential discharges	22	24	1/21 (4.8%)	19/21 (90.5%)	Pre-rehab.: 21/22 (95.5%); Post-rehab.: 6/22 (27.3%)	77.3
Chan, 2020	UK	Community rehab. team	Investigate individual characteristics that predict successful progress	Obs., retro.	All transfers to service within study period	193	51	NR	45/193 (23.3%)	NR	100
Dalum, 2018	Denmark	Community rehab. team	Investigate effectiveness of IMR compared to TAU in Denmark	RCT	Schizophrenia and bipolar disorder patients at one of three community services	198 (IMR=99; TAU=99)	12	NR	NR	Mean number of admissions (SD): IMR=0.6 (1.1), TAU=0.6 (1.7)	96.2
D'Avanzo, 2004	Italy	Supported accom.	Identifying factors that predict discharge from res. services	Obs., prosp.	All patients of res. facilities in Lombardy during study period	1792 (low-staffed=133; medium-staffed=126; high-staffed=1042; res. care=491)	13	49/1792 (2.7%)	191/1792 (10.7%)	NR	100

First author, Year published	Coun-try	Primary setting	Main aim	De-sign	Sample selection	N (by group)	Follow-up (FU) in months	Ratio (%) moved to higher supportive setting	Ratio (%) moved to lower supportive setting	Inpatient service use	Qual-ity rating
**Contemporary rehabilitation studies (continued)**											
de Girolamo, 2014	Italy	Supported accom.	To describe res. facility patients and investigate associations with discharge at one-yr FU	Obs., prosp.	All patients staying in 23 medium-long-term res. facilities provided by St John of God Order with a primary psychiatric diagnosis	403	12	6/403 (1.5%)	64/403 (15.9%)	NR	86.4
de Girolamo, 2016	Italy	Supported accom.	Compare demographics and outcomes of persons in services with history of violence vs controls	Obs., prosp.	Patients living in res. facilities at four different sites	139	12	1/139 (0.7%)	20/139 (14.4%)	NR	81.8
de Mooij, 2016	Nethe-rlands	Supported accom.	Track changes in residence and care settings	Obs., prosp.	Random selection from patients treated by outpatient teams, sheltered housing or inpatient care	59	72	NR	NR	NR	100
Fardig, 2011	Swe-den	Comm-unity rehab. team	Evaluate effectiveness of IMR program	RCT	Patients from study sites meeting inclusion criteria	41 (IMR=21; TAU (psych outpatient rehab. centre)=20)	12	NR	NR	12m at baseline: IMR = 4/21 (19.0%); TAU = 5/20 (25.0%); 12m at 21m FU: IMR = 0/19 (0%); TAU = 2/19 (10.5%)	88.5
Hanrahan, 2001	US	Supported accom.	Examining resident satisfaction with community integrated living arrangement facilities and compare hospital days before and after	Obs., prosp.	Resident at randomly selected community integrated living arrangement facility, with at least one-yr residence	74 (Integrated=43; Continuous=31)	12	NR	NR	Mean = 5.3 (SD 17) in first yr at community living arrangement facilities vs 47.7 (SD 103) in 1yr prior to facility	72.7
Incedere, 2019	Tur-key	Comm-unity rehab. team	Investigate outcomes of a hybrid case management model for patients with schizophrenia	Obs., prosp.	Patients living with caregivers and unable to live independently, treated with case management	30	24	NR	NR	24-month pre mean number of admissions = 1.33 (SD 1.06); 24-month during treatment/FU = 0.23 (0.56)	59.1

First author, Year published	Coun-try	Primary setting	Main aim	De-sign	Sample selection	N (by group)	Follow-up (FU) in months	Ratio (%) moved to higher supportive setting	Ratio (%) moved to lower supportive setting	Inpatient service use	Qual-ity rating
**Contemporary rehabilitation studies (continued)**											
Jensen, 2019	Den-mark	Comm-unity rehab. team	Effectiveness of IMR in Denmark	RCT	Patients at three community services	198 (IMR=99; TAU 99)	12	NR	NR	Mean difference in hospital days reported only (not means of groups) between IMR and TAU = 19.4 (95% CI: -0.76-0.5)	100
Kavanagh, 2009	Ire-land	Hospital rehab. unit	Describe characteristics and progress of inpatient rehab. patients	Obs., retro.	First patients admitted to service	50	60	0	17/46 (37.0%)	NR	83.3
Killaspy, 2013	UK	Hospital rehab. unit	Investigate five-yr outcomes for rehab. service users	Obs., retro.	All patients at selected services were potentially approached for participation	141 (47 inpatient unit, 44 community unit, 50 supported accom)	60	41/124 (33.1%)	50/124 (40.3%) (Inpatients: 19/47 (40.3%); Community & supported accom.: 31/94 (33.0%)	NR	95.5
Killaspy, 2016	UK	Comm-unity rehab. unit	Longitudinal outcomes for services users of inpatient rehab. units	Obs., prosp.	All patients of selected services	362 (339 FU)	12	0	187/339 (55.2%)	NR	100
Killaspy, 2019	UK	Supported accom.	Investigate 30-month outcomes for users of supported accommodation	Obs., prosp.	Randomly selected services users from nationally representative, randomly selected services	619 (159=RC; 251=SH; 209=FO)	30	NR	243/586 (41.5%) (RC 15/146 (10.3%); SH 96/244 (39.3%); FO 132/196 (67.3%))	Proportion with any admission during FU: 110/586 (18.8%): RC 27/146 (18.5%); SH 60/244 (24.6%); FO 23/196 (11.7%)	100
King, 2000	UK	Comm-unity rehab. unit	Investigate variables predictive of difference in outcome and explore patterns of change over time	Obs., prosp.	All residents during study period	20	26	5/20 (25.0%)	8/20 (40.0%)	NR	59.1

First author, Year published	Coun-try	Primary setting	Main aim	De-sign	Sample selection	N (by group)	Follow-up (FU) in months	Ratio (%) moved to higher supportive setting	Ratio (%) moved to lower supportive setting	Inpatient service use	Qual-ity rating
**Contemporary rehabilitation studies (continued)**											
Lascorz, 2018	Spain	Supported accom.	Evaluate 'evolution' of patients in first 10 years of a res. service	Obs., retro.	Residents with minimum of 2yr stay at service	93	60	14/93 (15.1%)	19/93 (20.4%)	Mean number of hospital days at one-yr prior to residence = 101, during 1st yr = 6.2, 2nd yr = 8.6, 3rd yr = 7.2, 4th yr = 2.3, 5th yr = 6.8, 6th yr = 9.5, 7th yr = 12.9, 8th yr = 12.1, 9th yr = 10.1, 10th yr = 14.1 (n not reported for each time-point)	54.5
Lee, 2009	US	Supported accom.	Investigate individual and housing characteristics associated with positive and negative programme discharge	Obs., retro.	Residents with minimum of 6m stay at service	237	30	41/237 (17.3%)	32/237 (13.5%)	NR	63.6
Lichten-berg, 2008	Israel	Comm-unity rehab. team	Assess effectiveness of clinical case management in 'revolving door' patients compared to TAU and no treatment	RCT	Patients identified via the national psychiatric case registry as having had three admissions during 1996-97.	370 (CCM=122; TAU=95; NT=153)	12	NR	NR	Proportion with any admission during FU: 248/370 (67.0%) (CCM=87/122; TAU=75/95; NT=86/153)	80.8
Macphers on, 2017	UK	Hospital rehab. unit	Evaluate outcomes of two inpatient recovery units	Obs., retro.	All patients at selected services	43	12	5/43 (11.6%)	38/43 (88.4%)	NR	81.8
Malinovsk y, 2013	US	Supported accom.	Track a range of recovery indicators after an organisation-wide implementation of the recovery model	Obs., prosp.	All residents of service by provider	627	12	NR	NR	Does not distinguish psychiatric and physical hospitalisation. Mean (SD) number of hospital days for month before recovery model = 9.79 (26.68) and month after 5.52 (19.74). Total number of hospital days one-yr pre recovery model = 4994, and one-yr post = 2970	95.5
Muir, 2008	Aust-ralia	Supported accom.	Evaluate Mental Health Housing and Accommodation Support Initiative	Obs., prosp.	Resident at service during two-yr study period	110	12	NR	NR	For n=67: Mean number of hospitalised days per person per year pre-HASI = 88.7 and during-HASI = 16.8. Mean number of days hospitalised per admission pre-HASI = 29.9 and during-HASI = 6.7	63.6

First author, Year published	Coun-try	Primary setting	Main aim	De-sign	Sample selection	N (by group)	Follow-up (FU) in months	Ratio (%) moved to higher supportive setting	Ratio (%) moved to lower supportive setting	Inpatient service use	Qual-ity rating
**Contemporary rehabilitation studies (continued)**											
Norden-toft, 2012	Den-mark	Supported accom.	Exploring the population residing in psychiatric supported housing facilities in Denmark	Obs., retro.	From national register: resident at service with previous psychiatric contact, matched with controls without any history of supported accommodation	5722=cases; 28,085=control	12	NR	NR	Mean hospitalised days in one-yr preadmission to residence / matching: 167 for both groups; one-yr post admission to residence / matching: 26.9 (cases) and 101.6 (control)	95.5
Parker, 2020	Aust-ralia	Comm-unity rehab. unit	To investigate predictors of positive outcomes for users of community care units	Obs., retro.	Discharge from service with one-yr pre-admission and one-yr post-discharge administrative records	501	12	NR	NR	Mean number of hospital days one-yr pre-admission mean = 101.54 (SD 113.01); one-yr post-discharge mean = 70.39 (SD 118.33)	100
Sakiyama, 2002	Japan	Supported accom.	Identifying conditions where use of support house is effective	Obs., prosp.	Discharged from service between April 1992 and October 1998	55	38	25/55 (45.5%)	30/55 (54.5%)	NR	77.3
Shadmi, 2018	Israel	N/A	To investigate whether PROMs can be used to predict rehospitalisations for people with schizophrenia	Obs., retro.	All schizophrenia patients in surveyed areas (central and north Israel) who have used psychiatric rehab. services were approached	2842	12	NR	NR	Proportion with any admission during FU: 379/2842 (13.3%)	100
Tan, 2017	Sing-apore	Comm-unity rehab. team	Compare IMR with TAU on symptoms, rehospitalisations and social functioning	RCT	Patients referred to service who met study criteria (including >2 admissions in previous yr)	50 (IMR=25; TAU=25)	24	NR	NR	Mean number of hospital days at 12-month FU: IMR = 0.00 (SD 0.00); TAU = 25.68 (24.11); and at 24-month FU (12 months post treatment): IMR = 0.08 (SD 0.40); TAU = 21.64 (20.01)	73.1

First author, Year published	Coun-try	Primary setting	Main aim	De-sign	Sample selection	N (by group)	Follow-up (FU) in months	Ratio (%) moved to higher supportive setting	Ratio (%) moved to lower supportive setting	Inpatient service use	Qual-ity rating
**Contemporary rehabilitation studies (continued)**											
Wong, 2008	US	Supported accom.	Investigate patterns and reasons for residents leaving supported independent living	Obs., retro.	All residents	452	21	63/452 (13.9%)	52/452 (11.5%)	Proportion with any admission during FU: 86/452 (19.0%)	85.0
**Services for homeless people with long term and complex mental health problems**											
Aubry, 2015	Can-ada	Supported accom.	Effectiveness of Housing First (HF) compared to TAU	RCT	People with severe mental illness who were homeless or precariously housed. Referral into study by health and social service agencies	950 (HF=469; TAU=481)	12	HF= 60/450 (13.3%); TAU= 121/406 (29.8%)	NR	NR	100
Aubry, 2016	Can-ada	Supported accom.	Effectiveness of HF with assertive community treatment compared to TAU (extension of Aubry 2015)	RCT	People with severe mental illness who were homeless or precariously housed. Referral into study by health and social service agencies	950 (HF=469; TAU=481)	24	NR	NR	Number of days hospitalised across both groups reduced by 62%, a similar reduction for both groups (NR by group)	100
Gilmer, 2010	US	Supported accom.	Recovery, service utilisation and cost outcomes of Full Service Partnership (FSP)	Obs., retro.	Admission between October 2006 and December 2007	363 (FSP=209; Control=154)	12	NR	NR	NR	90.9

First author, Year published	Coun-try	Primary setting	Main aim	De-sign	Sample selection	N (by group)	Follow-up (FU) in months	Ratio (%) moved to higher supportive setting	Ratio (%) moved to lower supportive setting	Inpatient service use	Qual-ity rating
**Services for homeless people with long term and complex mental health problems (continued)**											
Gilmer, 2014	US	Supported accom.	Compare service use and costs of FSP clients with matched controls	Obs., retro.	Participants identified from Health Records database, enrolled on FSP between Jan 2005 and June 2009. Matched to participants on demographics and clinical characteristics, and health service use	20462 (FSP= 10231; Control= 10231)	12	NR	NR	Mean (standard error) number of hospital days for: FSP: 12-month pre = 12.2 (0.3), 12-month post = 7.8 (0.2); Control: 12-month pre = 11.6 (0.3), 12-month post = 7.2 (0.2)	95.5
Gilmer, 2014	US	Supported accom.	Fidelity of FSP to HF and res. outcomes	Obs., retro.	Patients enrolled on FSP for at least 180 days between January 2005 and June 2009	6584 (High fidelity=1858, Moderate=3481, Low=1245)	12	NR	NR	NR	100
Gulcur, 2003	US	Supported accom.	Comparing HF with supported housing (SH)	RCT	Recruited from streets and hospitals	225 (HF=99; SH=126)	24	NR	NR	NR	65.4
Lipton, 2000	US	Supported accom.	To study long-term effectiveness of housing approaches for homeless persons with severe mental illness	Obs., retro.	Residence at high/moderate/low -intensity service during study period	2937 (High=873, Moderate=540, Low=1524)	30	NR	NR	NR	85.0
McHugo, 2004	US	Supported accom.	Comparing two approaches to linking housing with mental health services (integrated: housing and support provided by the same service, and parallel: provided by separate services)	RCT	Recruited from various sources. Current or risk of homelessness and severe mental illness	121 (Integrated=61, Parallel=60)	18	NR	NR	NR	80.8

First author, Year published	Coun-try	Primary setting	Main aim	De-sign	Sample selection	N (by group)	Follow-up (FU) in months	Ratio (%) moved to higher supportive setting	Ratio (%) moved to lower supportive setting	Inpatient service use	Qual-ity rating
**Services for homeless people with long term and complex mental health problems (continued)**											
O'Campo, 2016	Can-ada	Supported accom.	Impact of HF on housing, justice system, health service use and health outcomes	RCT	Participants at the Toronto site from the At Home/Chez Soi study - homeless or precariously housed at recruitment	241 (HF=113; TAU=128)	24	NR	NR	Mean (95% CI) of days in psychiatric hospitals, amongst those hospitalised: HF= 65.8 (40.9-105.8); TAU= 168.4 (98.1-289.0)	88.5
Stefancic, 2007	US	Supported accom.	Access and retention of housing in HF and TAU	RCT	Referral to study for randomisation to HF or TAU (chronic shelter users with psychiatric disabilities)	260 (HF=209; TAU=51)	47	NR	NR	NR	58.3
Tsem-beris, 2003	US	Supported accom.	Present initial results for a longitudinal study evaluating housing stability for HF programme	RCT	Referral to study, mainly by services in contact with chronic homelessness and severe mental illness	225 (HF=99; Control=126)	6	NR	NR	NR	73.1
Tsem-beris, 2004	US	Supported accom.	Examine longitudinal effects of HF (extension of Tsemberis, 2003)	RCT	Two sub-samples: 1. Street homeless 2. Psychiatric hospital (and homeless before hospitalisation)	225 (HF=99; Control=126)	24	NR	NR	NR	65.4
van Kranen-burg, 2020	Nethe-rlands	Hospital rehab. unit	Investigate relationship between clinical changes during treatment and long-term outcomes from SuRe (Sustainable Residence)	Obs., retro.	All admissions to SuRE, with minimum four-yr length of admission by study end (Jan 2015)	165	48	26/165 (15.8%)	70/165 (42.4%)	NR	86.4

First author, Year published	Coun-try	Primary setting	Main aim	De-sign	Sample selection	N (by group)	Follow-up (FU) in months	Ratio (%) moved to higher supportive setting	Ratio (%) moved to lower supportive setting	Inpatient service use	Qual-ity rating
**Deinstitutionalisation programmes**											
Baloush-Kleinman, 2003	Israel	Comm-unity rehab. unit	Outcomes of a deinstitutionalisation programme in Israel	Obs., retro.	All admissions to service	205	60	16/205 (7.8%)	138/205 (67.3%)	Proportion with any admission during FU: 16/205 (7.8%)	50.0
Barbato, 2004	Italy	Supported accom.	Reporting four-yr outcomes of all patients discharged from hospital to community residence	Obs., prosp.	All patients discharged from hospital to community residence	163	42	NR	NR	Proportion with any admission during FU: 35/163 (21.5%)	72.7
Barthol-omew, 2018	US	Comm-unity rehab. unit	Evaluation of a programme for long-stay patients difficult to discharge to community	Obs., retro.	All admissions within first yr of service opening	22	7	5/22 (22.7%)	11/22 (50.0%)	NR	50.0
Chan, 2007	Japan	Supported accom.	Effect of supported group residence compared to continued long-stay hospital admission	RCT	Patients in a private psychiatric hospital which met study criteria	28 (supported group residence=14; continued admission=14)	24	NR	NR	Proportion with any admission during FU: 3/14 (21.4%)	84.6
Chopra, 2011	Aust-ralia	Supported accom.	Investigate long-term outcomes for ex-long-stay patients who moved to a residential psychiatric service	Obs., retro.	All patients who moved to Footbridge Community Care Unit (CCU)	18	84	NR	1/18 (6%)	NR	68.2
Duurkoop, 2003	Nethe-rlands	Supported accom.	13-yr FU of patients discharged from long-stay hospital to two levels of community residence	Obs., prosp.	All clients at the two study sites (Kempering and Surinemeplain)	174 (Kemp. =84; Surin.=90)	121	7/174 (4.0%) (Kemp.=5/84 (6.0%); Surin.=2/90 (2.2%))	16/174 (9.2%) (Kemp.=15/84 (17.9%); Surin.=1/90 (1.1%))	NR	40.9

First author, Year published	Coun-try	Primary setting	Main aim	De-sign	Sample selection	N (by group)	Follow-up (FU) in months	Ratio (%) moved to higher supportive setting	Ratio (%) moved to lower supportive setting	Inpatient service use	Qual-ity rating
**Deinstitutionalisation programmes (continued)**											
Gamble, 2011	US	Hospital rehab. unit	Evaluation of a preparation for discharge programme	Obs., prosp.	Long-stay patients at several hospitals, did not select people with risk issues or complex needs	10	24	0	8/10 (80%)	NR	43.8
Hallam, 2002	UK	Comm-unity rehab. unit	Cost effectiveness of rehab. services for difficult-to-place long-stay patients	Obs., prosp.	Difficult-to-place patients from Friern Hospital moved to one of four rehab facilities	67	60	6/56 (10.7%)	24/56 (42.9%)	NR	63.6
Hobbs, 2000	Aust-ralia	Supported accom.	Investigating outcomes of ex-long stay patients discharged to community res. services	Obs., prosp.	All patients discharged from long-stay hospital (closure) to community residences	43	24	7/42 (16.7%)	3/42 (7.1%)	Proportion with any admission during FU: 20/43 (46.5%)	72.7
Hobbs, 2002	Aust-ralia	Supported accom.	Six-yr follow-up of ex-long stay patients (extension to Hobbs, 2000)	Obs., prosp.	All patients discharged from long-stay hospital (closure) to community residences	47	72	7/44 (15.9%)	26/44 (59.1%)	NR	59.1
Macphers on, 2004	UK	Supported accom.	Examine treatment outcomes of hostels in Gloucester	Obs., retro.	All patients admitted to any of the four hostels from open (between 1983 and 1993) and 1999	58	120	21/58 (36.2%)	10/58 (17.2%)	Proportion with any admission during FU: 19/58 (32.8%)	81.8
McCrone, 2006	UK	Comm-unity rehab. unit	Compare the service use and costs of 'difficult to place' patients discharged from Warley and Friern Hospital	Obs., prosp.	Difficult to place' patients from both hospitals	84 (Friern=63; Warley=21)	12	NR	NR	Proportion with any admission during FU: Friern: 5/63 (7.9%); Warley: 3/21 (14.3%)	86.4

First author, Year published	Coun-try	Primary setting	Main aim	De-sign	Sample selection	N (by group)	Follow-up (FU) in months	Ratio (%) moved to higher supportive setting	Ratio (%) moved to lower supportive setting	Inpatient service use	Qual-ity rating
**Deinstitutionalisation programmes (continued)**											
Meehan, 2011	Aust-ralia	Supported accom.	Evaluate clinical and social outcomes of a group discharged from long-stay hospitals into supported accom.	Obs., prosp.	All patients discharged from long-stay hospital to community residences	181	84	NR	NR	Proportion with any admission during FU: 112/181 (60.2%)	77.3
Noda, 2004	Japan	Hospital rehab. unit	Evaluating outcomes of an inpatient rehab. discharge programme	Obs., retro.	Patients enrolled on programme	224	84	47/224 (21.0%)	172/224 (76.8%)	NR	68.2
Ryu, 2006	Japan	Supported accom.	Evaluate outcomes of long-stay patients discharged to residential facility	Obs., prosp.	All patients transferred to Sasagawa Village	78	24	0	0	Proportion with any admission during FU: 4/78 (5.1%)	81.8
Tanioka, 2013	Japan	Hospital rehab. unit	Examine factors associated with discharge from long-stay hospital enrolled in a rehab. programme	Obs., retro.	All patients enrolled in programme with >1yr inpatient admission	70	12	NR	NR	NR	70.0
Trauer, 2001	Aust-ralia	Comm-unity rehab. unit	To follow all patients admitted to CCU from long-stay ward and investigate 1yr outcomes	Obs., prosp.	All patients on long-stay ward nominated for CCU	125	12	14/99 (14.1%)	4/99 (4.0%)	Proportion with any admission during FU: 11/70 (15.7%)	54.5
Trieman, 2002	UK	Hospital rehab. unit	Study long-term outcome of long-stay inpatients regarded unsuitable for community placement	Obs., prosp.	'Difficult-to-place' long-stay patients	72	60	0	29/72 (40.3%)	NR	81.8

Accom. = accommodation. ACT = assertive community treatment. BH = boarding home. BPR = Boston Psychiatric Rehabilitation. CBT = cognitive behavioural therapy. CCM = clinical case management. CCU = community care unit. DTP = day treatment programme. FU = follow-up. FSP = Full Service Partnership. HF = Housing First. IMR = Illness Management and Recovery. Kemp. =Kempering. NR = not reported. Obs. = observation study. Prosp. = prospective study. QoL = quality of life. RCT = randomised controlled trial. Rehab. = rehabilitation. Retro. = retrospective study. Res. = residential. SH = supported housing. SuRe = Sustainable Residence. Surin. = Surinemeplain. TAU = treatment as usual. Yr = year.
